# Porcine Deltacoronavirus Occurrence in the United States Breeding Herds since Its Emergence in 2014

**DOI:** 10.3390/v16030445

**Published:** 2024-03-13

**Authors:** Mariana Kikuti, Catalina Picasso-Risso, Cesar A. Corzo

**Affiliations:** 1 Department of Veterinary Population Medicine, University of Minnesota, Saint Paul, MN 55108, USA; mkikuti@umn.edu (M.K.);; 2 Department of Large Animal Clinical Sciences, Michigan State University, East Lansing, MI 48824, USA

**Keywords:** Porcine Deltacoronavirus, epidemiology, swine health, disease occurrence, incidence

## Abstract

PDCoV, an enveloped RNA virus, causes atrophic enteritis in neonatal piglets, leading to diarrhea, malabsorption, dehydration, and death. The study aims to fill the gap in the current epidemiological information about PDCoV in the U.S. pig population after its emergence in 2014. Data from the Morrison Swine Health Monitoring Project (MSHMP) between January 2015 and December 2023 were analyzed, representing approximately 60% of the U.S. breeding herd. Participating herds report weekly PDCoV health status. In total, 244 PDCoV outbreaks occurred in 186 sites from 22 production systems across 16 states. Case counts peaked during winter, and incidence ranged from 0.44% in 2017 to 4.28% in 2023. For sites that experienced more than one PDCoV outbreak during the study period, the interval between outbreaks was a median of 2.11 years. The South and Midwest regions reported the majority of cases. In 2017, a shift in the spatial distribution of cases from the Midwest to the South was observed. The findings underscore the importance of continued monitoring and strengthened control measures to mitigate the impact of PDCoV in U.S. breeding herds.

## 1. Introduction

Porcine *Deltacoronavirus* (PDCoV) is one of the four genera of viruses belonging to the Orthocoronavirinae subfamily which is a member of the *Coronaviridae* family of viruses. The *Deltacoronavirus* genus has three subgenera, *Andecovirus*, *Buldecovirus*, *Herdecovirus*. Within the *Buldecovirus* subgenus there are five species, one of them is known as the Coronavirus HKU15. This enveloped RNA virus with an approximate genome of 25–26 kb was detected in 17 (10.1%) out of 169 rectal swabs from pigs during a 4.5-year surveillance program in Hong Kong between February 2007 and June 2011 [1]. The origins of this virus are currently not fully understood; however, the same group of researchers who conducted the surveillance project compared complete HKU15 genome sequences with other viruses found during the study and concluded that a virus originating from sparrows shared 90% identity suggesting a potential recent spillover from birds to pigs [1]. At the time of its discovery, the clinical impact of this virus at the pig level was unknown.

In February 2014, PDCoV emerged in the United States (US) as the country was still managing the recent emergence of another coronavirus (i.e., Porcine Epidemic Diarrhea virus—PEDv). PDCoV-positive breeding farms were undergoing outbreaks of acute watery diarrhea in sows and suckling pigs, but the pre-weaning mortality rate did not have the severity of the recent PEDv outbreaks. During the diagnostic investigation, specimens sent to the veterinary diagnostic laboratories for testing yielded negative results for PEDv and other common baby pig enteric pathogens such as Transmissible gastroenteritis virus or Rotaviruses. These results prompted diagnosticians to develop a molecular assay to test for *Deltacoronaviruses* which detected the virus in samples of pigs with diarrhea [2]. At this stage, the widespread nature of this emerging virus was unknown in the U.S. and data from the Animal Disease Diagnostic Laboratory of the Ohio Department of Agriculture stated that 109 (25%) out of the 435 samples tested between 7 February 2014 and 9 April 2014 tested positive for PDCoV. Most of these samples originated from nine pig-producing states [3]. Furthermore, at the University of Minnesota Veterinary Diagnostic Laboratory, of the 293 porcine samples (comprising fecal swabs, fecal samples, saliva, intestinal homogenate, vomit, feed, and environmental samples) collected between 6 January 2014 and 27 February 2014, that were tested for PDCoV, 89 (30%) were positive, with the intestines and feces representing more than half of the positive samples [4]. On the 30 April 2014, the Novel Swine Enteric Corona Virus Disease Testing Summary Report prepared by the National Animal Health Laboratory Network (NAHLN) reported that a total of 13 states of the 24 surveyed had at least one positive RT-PCR result for PDCoV [5], indicating that the virus had been disseminating rapidly and without the industry’s knowledge. Whole genome sequencing of samples from different states reached a similar conclusion: that the recently emerged virus had a high nucleotide similarity with the HKU15 virus described in Hong Kong [2,4,6].

The pathogenesis of this virus was poorly understood at the time. Therefore, gnotobiotic 10-to-14-day-old pigs were inoculated with PDCoV. All infected pigs were shedding the virus via their feces, and developed watery diarrhea and vomiting by 21–24 h post inoculation. The virus caused severe atrophic enteritis throughout the intestine but the jejunum and ileum represented its primary infection sites, presenting with lesions that resembled Porcine Epidemic Diarrhea (PED) [7]. Recently, a study was conducted to assess the clinical signs, shedding, and immune response dynamics of PDCoV in older pigs under experimental conditions. The seven-week-old orally challenged pigs presented an active viral shedding peak at 10 days post-inoculation. Interestingly, no clinical signs suggestive of an enteric disease were observed in the inoculated pigs which may explain how subclinically infected pigs could have been moved between sites without suspecting that there was an infectious disease [8]. 

Field studies related to the epidemiology of PDCoV are scarce. It is known that when breeding herds become infected with PDCoV swine practitioners and producers are able to eliminate it through a load–close–expose approach (loading the sow herd with gilts followed by temporarily ceasing replacement gilt introduction and mass exposing the herd to the live virus) as it has been used for other swine infectious diseases. For example, a breeding stock company, which was involved in a significant outbreak in the U.S., reported that 47% of their multiplication breeding herds were infected with PDCoV. The company successfully eradicated PDCoV from their system with the breeding herds requiring an average of 14.6 weeks from the introduction of the virus to consistently begin weaning RT-PCR negative pigs [9]. Another study focused on estimating the manure pit prevalence of PDCoV in Minnesota [10]. The virus was identified in 19 (6.3%) out of 300 pits tested, with a median cycle threshold value of 34.49, ranging from 28.65 to 39.75. However, researchers were unable to ascertain the viability of the virus or for how long it remains detectable in these pits. These studies contributed to our understanding of the epidemiology of this disease but there are currently multiple gaps that need to be addressed as the current situation of PDCoV in the U.S. is largely unknown. Therefore, the objective of this study is to characterize the PDCoV occurrence in the U.S. breeding herd population since its emergence.

## 2. Materials and Methods

### 2.1. Data Source

Data were provided by the Morrison Swine Health Monitoring Project (MSHMP), a Swine Health Information Center (SHIC)-funded project. This voluntary project was established in 2011 and is aimed at building capacity to respond in a timely manner should a new infectious disease agent emerge. Therefore, the project curates and analyzes health information from U.S. breeding herds to enhance preparedness, building trust and collaboration, while also monitoring endemic diseases and generating objective metrics for disease occurrence and elimination. Briefly, upon production system enrollment, companies share information such as farm location, herd size, air filtration status, premises identification number, current and, if available, retrospective health status data. The project obtains weekly health status of participating herds for high impact infectious diseases and pathogens such as PDCoV, PED, and Porcine Reproductive and Respiratory Syndrome (PRRS). Information obtained from participating systems relates to whether herds have had an outbreak, reached stability (e.g., weaning PCR negative pigs), or even eliminated the infectious disease agent. The swine production companies and swine veterinary clinics currently participating in MSHMP represent over 60% of the U.S. breeding herd. As such, the monitored herds provide a representative population denominator for describing disease occurrence in the U.S. In 2013 when PED emerged, MSHMP played a pivotal role in that it was able to obtain voluntary data and rapidly generate estimates of disease occurrence and monitored the rate at which herds were eliminating the virus. 

Overall, a PDCoV outbreak is characterized by a farm encountering a sudden surge in piglets with watery diarrhea, resulting in elevated preweaning mortality, along with a positive PDCoV RT-PCR result from tests on affected piglets. However, the decision to conduct symptomatic surveillance and testing was left to the discretion of each veterinarian within the participating production systems. These veterinarians are encouraged to report any detected outbreaks to the MSHMP. Data for this study included farm characteristics and demographics (i.e., U.S. region, sow herd size, and air filtration type when available), and PDCoV outbreak occurrence between 1 January 2015 and 31 December 2023. 

### 2.2. Descriptive Analyses

Data assembly, cleaning, and visualization were performed in STATA 18 [11]. An overall count of the PDCoV outbreaks reported per month was represented by an epidemiological curve generated from January 2015 to December 2023. Farm characteristics for the herds that have reported at least one case were described by basic summary statistics. Case locations were described according to U.S. Census regions into West, Midwest, Northeast, and South [12]. Yearly cumulative incidence was calculated using the number of breeding herds reporting either PRRS, PEDv, or PDCoV statuses (i.e., sites that were sharing information for at least one of the main diseases monitored by MSHMP) as the denominator and reported cases as the numerator.

### 2.3. Ethics Statement

No ethical approval was required as this article describes data collected through routine veterinary diagnosis by the farms’ veterinarians as part of standard care.

## 3. Results

**Figure 1:**
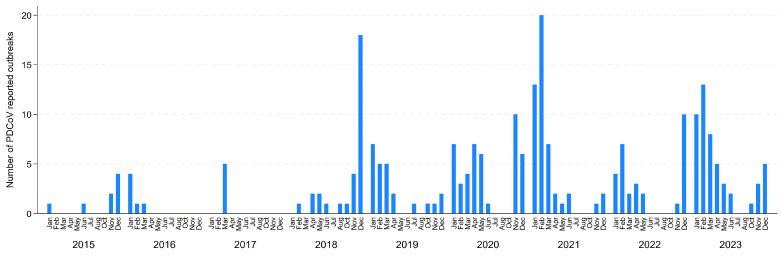
Number of herds that reported a PDCoV outbreak by month/year.

Throughout 2015–2023, the median number of sites monitored through MSHMP was 1166, ranging from 1062 in 2015 to 1187 in 2020. Since January 2015, a total of 244 PDCoV outbreaks have been reported to MSHMP and these originated from 186 sites from 22 production systems located in 16 U.S. states. During the entire period, one hundred and forty herds reported one PDCoV outbreak, thirty-six herds reported PDCoV twice, eight reported it three times, and two herds reported PDCoV outbreaks four times. For sites that experienced more than one PDCoV outbreak during the study period, the interval between outbreaks had a median of 2.11 years (interquartile range—IQR: 1.7–2.80, ranging from two months to almost 5 years). Figure 1 summarizes the monthly number of PDCoV cases over time in which a cyclical pattern can be observed as cases are more frequently reported during the fall and winter seasons. Although in January 2015 only one PDCoV case was reported, transmission became more frequent but still remained low up until late 2018. Since then, we have observed a mean of 4.69 PDCoV cases per month or a median of 3 cases per month. Two larger peaks in PDCoV cases were observed in December 2018 and in February 2021, with 18 and 20 cases reported involving four and three production systems, respectively. In 2023, the peak in PDCoV cases reported also occurred in February, with 13 cases involving three production systems. The total number of PDCoV cases reported per year was eight in 2015, six in 2016, five in 2017, thirty in 2018, twenty-four in 2019, forty-four in 2020, forty-eight in 2021, twenty-nine in 2022, and fifty in 2023.

**Figure 2:**
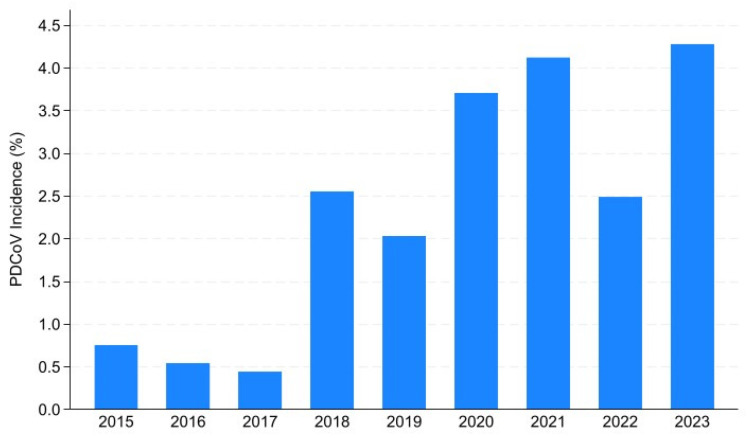
PDCoV yearly cumulative incidence in U.S. breeding herds between 2015 and 2023.

Amongst sites that reported a PDCoV outbreak, the median sow herd size was 3150 (IQR: 2350–4750). Information on air filtration was not available for 62.90% (*n* = 117) of the sites, while 29.03% (*n* = 54) of them were not filtered, and 8.06% (*n* = 15) were filtered (either partially or year-round). Most cases occurred in the South (*n* = 130, 69.89%) and the Midwest (*n* = 46, 24.73%), with the remaining cases located in the West (*n* = 3, 1.61%) and Northeast (*n* = 1, 0.54%). No location information was available for the remaining six sites that reported at least one PDCoV outbreak. Although all cases occurred in the Midwest in 2015 and 2016, cases from 2017 originated only from the South. The yearly cumulative incidence is shown in Figure 2, and ranged from 0.44% in 2017 to 4.28% in 2023.

## 4. Discussion

Unlike the rapid nationwide spread observed after the introduction of PEDv to the U.S., leading to a significant epidemic in 2014 with 40% cumulative incidence in breeding herds [13], PDCoV appears to have had a lower occurrence and limited ability to spread. In the first few years of PDCoV occurrence in the U.S., we detected cases mainly in the Midwest, comparable to the occurrence reported at the time, when most reported cases originated from Ohio, Illinois, Minnesota, Nebraska, and Iowa [2,4,6,14]. However, a noteworthy shift in the spatial distribution of cases occurred over time. Since then, most cases detected through the MSHMP surveillance originated from the South. 

We also found most peaks in PDCoV case counts occurred during the fall–winter months suggesting that even though cases are also reported in warmer months, there is a potential seasonality of PDCoV transmission. This is partially supported by an increased positivity rate in clinical samples collected from the Henan province in China during winter (54%) and spring (20%) [15]. This seasonality is also observed in PRRS and PED transmission in the U.S., where most cases occur during winter [16,17,18]. It is not fully understood why cases cluster during this time of the year and it is hypothesized that lower environmental temperatures may contribute to maintaining pathogens’ viability, and thus allow for indirect transmission.

Our voluntary monitoring project allowed us to estimate the PDCoV incidence in U.S. breeding herds as participating production systems and veterinary clinics reported the detection of the virus per their routine monitoring and standard care. However, no standardized classification of PDCoV status has been proposed that would allow us to estimate disease prevalence fluctuations through time. Moreover, the absence of universally adopted standardized criteria for declaring a herd negative post-outbreak hampers our ability to have a comprehensive understanding of an outbreak’s duration. In fact, two herds reported a new PDCoV outbreak only about 2 months after their previously reported outbreak. Efforts to standardize monitoring and classifying a herd as negative after an outbreak should be made if the goal is to better understand the disease dynamics. For example, PRRS health status takes into account the monitoring/testing frequency and intermittent positive results that may occur towards disease elimination [19]. 

PDCoV occurrence in the U.S. has been mostly described as the percentage of positive submissions. In 2014, about 25–30% of submissions from clinically suspected cases (diarrheal disease) were positive from two Midwest laboratories [3,4], while nationwide, the positivity rate in PDCoV submissions was 2.8% between 2014 and 2016 in 17 laboratories [20]. More recently, positivity rates have been reported to be between 1% and 7.5% [21], with peaks in positivity rates mostly coinciding with the case counts and incidence peaks described here. While most of these reports present a higher positivity rate than the cumulative incidence we found in their corresponding time frames, it is important to remember that this is expected since laboratory submissions will more frequently represent clinically relevant cases in which the probability of testing positive is higher than in the general population. Moreover, our base population includes solely breeding herds, while the laboratory submissions might also include growing pig herd cases. Additionally, the same population could be sampled and tested more than once during the outbreak, which can lead to overestimations in submissions. The cumulative incidence estimation in our project, however, tends to more accurately represent the overall health status of the U.S. breeding herd as a whole.

The main limitation of the presented data is that is it based on voluntary reporting from participating production systems that monitor their herds for the main swine diseases affecting the U.S. breeding herds. However, they might prioritize the reporting of more prevalent diseases that heavily impact the industry, such as PRRS and PED. Thus, it is possible that PDCoV cases are underreported. Still, laboratory testing for PDCoV is relatively common in the context of diarrheal diseases. This involves a triplex RT-PCR for the simultaneous detection of PEDv, PDCoV, and TGEV, routinely conducted to increase the likelihood of case detection through symptomatic surveillance, given the similar clinical manifestations between these three enteric diseases.

Overall, we found that, even though PDCoV occurs at a much lower frequency than other important swine diseases in the U.S., such as PRRS and PED, it is still very much present in the U.S. breeding herd as cases continue to be reported each year. In fact, PDCoV incidence has increased when compared to earlier years. These results show that PDCoV monitoring is still important, and control measures need to be strengthened to limit the spread and impact of the disease.

## Data Availability

Data used in this article are not publicly available due to privacy restrictions by the production systems participating in this project. Data are available on request to the corresponding author, provided consent from the production companies involved is given.
